# Patients' Contributions to Hospitals

**Published:** 1888-06-23

**Authors:** W. Burdett-Coutts


### PATIENTS' CONTRIBUTIONS TO HOSPITALS.

BY MR. W. BURDETT-COUTTS, M.P.

*(Concluded from p. 178.)*

### IRISH HOSPITALS.

FROM Ireland fifteen replies have been received. At the Belfast Royal Hospital, with 200 beds, in-patients' payments amounted to £1,070, against an expenditure of about £7,000. With the exception of two special beds, they are treated in general wards. Their payments range from 5*s.*, 7*s.*, 10*s.* 6*d.*, 14*s.*, 15*s.*, up to 21*s.* per week. The Board of Management meets once a-week to admit patients, and "if any doubts should arise as to the applicant's statement as to ability to pay, their clergyman, or other known person, is written to for information." It is worthy of notice, in connection with what I said at the beginning of this paper with regard to the employment of a surplus of funds, that the Belfast Royal has a branch hospital, called the Throne Hospital, with thirty beds. At the Belfast Skin Hospital, where the receipts from paying patients were £150, against an expenditure of about £350, their means are judged according to their "occupations, street, or locality in which they live." At the Cork Fever Hospital, those who can afford one guinea per week are treated in private wards; others, in general wards, pay according to their means, the maximum being ten shillings and sixpence per week. The secretary says:—

"It gives respectable people of small means a feeling of independence when entering the hospital, that they are not being treated for charity's sake, when otherwise they might insist on being treated at their homes and risk ensue to others."

At the Dublin Eye and Ear Institution, with thirty beds, all in-patients pay if they can afford it. The following interesting statement of accounts has been supplied by the Secretary:—

| | 1885. | 1886. | 1887. |
|---|---|---|---|
| In-Patients | £308 12 11 | £263 10 0 | £297 0 10 |
| Out-Patients | 106 3 0 | 111 1 0 | 116 11 6 |
| | £414 15 11 | £374 11 0 | £413 12 4 |
| Maintenance of— | | | |
| Patients | £417 15 9 | £340 4 5 | £380 13 4½ |
| Establishment | 375 14 5 | 372 15 3 | 554 7 9 |
| Management | 158 5 7 | 133 14 6 | 134 11 10 |
| Loss on composition in Munster Bank | 44 8 0 | | |
| | £993 3 9 | £846 14 2 | £1,069 12 0 |

He adds that he is

"Quite satisfied with the system which has been in use here for 15 years. While it makes the institution largely self-supporting, it is calculated to promote the spirit of self-reliance amongst the class who frequent it. It is considered only right that those who enjoy the benefit of the hospital should contribute according to their means towards its maintenance."

### ADELAIDE HOSPITAL, DUBLIN.

There is a point brought out in the reply from the Adelaide Hospital, Dublin, where the patients' payments for last year amounted to £840, to which I would call special attention. Patients are admitted by subscribers' letters, which state either the sum that the patient can afford to pay or that he is unable to pay the minimum sum charged. This latter statement illustrates what I think should be the first and perhaps the sole condition of the Pay System, viz., that the patient, after being asked to contribute what he can, should, if he states that he cannot contribute anything, be called upon to prove his inability to do so. We do not wish him to prove what he can, but what he cannot, pay.

### PROVIDENT DISPENSARIES.

I have found it impossible in this paper to deal, except in the form of the tables annexed, with the question of provident dispensaries. They unquestionably have an important bearing on the adoption of the Pay System in the out-patient departments of our great hospitals, and the financial results of contributors' payments are so large as to make the treasurer of any hospital glance with an envious eye at the system. Without venturing to express an opinion upon the special questions involved, I would say that it seems to me that it is not altogether outside of the range of human possibility that the whole Provident System may be overdone. At least, in the first instance, I should like to see it confined to the inevitable, then the contributor could never be defrauded of his results. But to the man who continues for weeks or for years to provide against sickness, sickness may never come. His hoarded provision is lost to him, and in these hard days few poor men can afford to save for other people. I think the system of providing against sickness should contain some element of alternative advantage to the subscriber if he is never sick; in fine, although I speak with some hesitation on the subject, I had rather see a good Pay System attached to the out patient department of a hospital than see that department transformed into a provident dispensary.

### COTTAGE HOSPITALS.

Lastly, there remains a class of hospitals which present many interesting and important points for consideration in connexion with this subject, and from which I have received so many valuable replies that I consider them well worth a separate paper. But the bearing that these hospitals have upon the adoption of the Pay System in London is more with respect to its principle than its practice. I have endeavoured throughout this paper to direct attention to the method by which the means of a patient may be ascertained, and the payment properly due from him to the hospital fixed with approximate fairness. Whatever method is adopted —and the less inquisitorial it is, in my opinion, the better—there is no doubt that all cottage hospitals are specially favoured in this respect. The smallness and constant intercommunication of the populations affected renders imposition difficult and a just apportionment of the payment to the patient's circumstances easy. Putting this aside, if we find that the system of paying patients works well in the country, and makes a valuable contribution to the funds of the hospital, there is all the more reason for imitating the example in London. I cannot understand, particularly in these times of great agricultural depression, how the country labourer, with wages varying from ten shillings to fourteen shillings a-week, should be better able to pay than the London artisan, clerk, and tradesman. I know that the perpetual relations between landowners and their labourers lead the former, in some cases, to pay for the labourers' treatment in the cottage hospital; if so, there is one little benefit left out of the feudal system. But is it possible that in all cases the Londoner, who is proud of having emancipated himself, many centuries since, from any such influences, is content to replace them with the charity of others, who do not know him and with whom he has no connexion? I am too good a Londoner myself to believe it of the great working masses of this City. I do not expect them suddenly to start off paying without inducement, example, or system; but I do believe that if the machinery which is so simple in country districts could be extended and fitted to the London population, the latter, seeing its far-reaching advantages to themselves and their children after them, would readily fall into the system; but of ninety-two replies from cottage hospitals I find that only seventeen do not take paying patients. Of the remainder the proportion of patients' payments to the total expenditure is in one case 75 per cent., in three cases 50 per cent., in one case 40 per cent, in five 30 per cent., in five 20 per cent., in sixteen 15 per cent., in seventeen 10 per cent., in three 5 per cent., and in four under 5 per cent. It will not be denied that these are very important contributions to the financial soundness of the various institutions. If they could be repeated in the great hospitals in London our difficulties would be nearly at an end.

Summary.

It now remains for me, before closing this paper, to gather up the various points which bear upon the Pay System as applicable to the great London Hospitals.

1. The Pay System is a recognised and sound element, universal in other countries, and largely practised, in various forms, both in large and small and in general and special hospitals in this country.

2. Large actual deficit at present, excluding legacies, which are unreliable.

3. Charitable contributions to hospitals are enormous, but are not able to support the present system, and with the constant growth of London will be less able every year; gradual but growing discouragement to contributors. No principle of self-help attraction in other charitable enterprise, exists in present system. It turns a hospital into an almshouse, and pauperises its patients.

4. The blot in the system. Incidence of charity is wrong. The hospitals are on a false economic basis.

5. The rectification of this will widen and multiply the benefits to the poor, and will give confidence to subscribers.

6. Why should Pay System exist largely in special hospitals, and not in general ones? I am not aware that Nature, in her choice of victims for special or general complaints, makes any selection based upon the means of the patient.

7. The Pay System means that every person should pay what he can.

(*a.*) Governors' letters are not the Pay System, unless they carry with them assurance from personal knowledge that patient is able to pay a certain sum, or unable to pay anything.

(*b.*) On the other hand, free letters to bodies of work-people, in answer to subscriptions from them, are the Pay System. The latter should carry a share in hospital government.

(*c.*) Free hospitals, strictly confined to patients who can pay nothing, would form part of a general Pay System.

8. The Pay System should, where possible, include a remunerative ward, such as Saint Thomas's Home.

9. For general patients a system of self-assessment, as carried out in America, would be best, *i.e.*, patient should be called upon to prove his inability to pay. This can be done through—

(*a.*) Subscribers having knowledge of the circumstances.

(*b.*) Visiting committees.

(*c.*) Clergyman or doctor.

(*d.*) In small hospitals, inquiries by secretary.

10. Fixed scale of graduated payment, according to class of ward occupied, would be possible. Patient would always prefer the best ward he could pay for.

11. Pay System should be applied to out-patient department (perhaps with provident system attached) as well as to in-wards.

12. Pay System must be universal to be completely successful. Universal Free Hospitals impossible.

13. Some adjustment between poor-law infirmaries and free wards of hospitals is necessary to prevent unfair preference to different portions of the same class, *i.e.*, the absolutely poor.

14. Pay System, as practised through workmen's contributions in solid communities in the North, or by adjusted payments, as in special and Cottage Hospitals, more difficult in sporadic and disbanded elements of London population; but where energetically tried, as in seamen's contributions to *Dreadnought* Hospital, and in Glasgow, Leeds, and Manchester, have been thoroughly successful. The former method represents its most important development in the North and should be earnestly tried in London.

15. Conclusion. The Pay System is based on the principle of self-help; adjusts the relations between that and eleemosynary assistance; affords skilled treatment to large numbers unable to procure it at home; extends the area of benefit to the poor; gives organic support to the hospital finances; and therefore every effort should be made to extend it in London.

